# Developing Educational Materials to Support Older Adults With Hypertension Management

**DOI:** 10.1093/geroni/igab046.882

**Published:** 2021-12-17

**Authors:** Wendy Rogers, Qiong Nie, Maurita Harris, Stacy Al-Saleh, Ysabel Beatrice Floresca

**Affiliations:** 1 University of Illinois Urbana-Champaign, Champaign, Illinois, United States; 2 University of Illinois at Urbana Champaign, Champaign, Illinois, United States; 3 University of Arizona, Tucson, Arizona, United States

## Abstract

A comprehensive approach to hypertension management requires medication adherence as well as more general health behavior changes. Our primary objective is to provide evidence-based and tailored education about hypertension, medications, and health self-management strategies with consideration for different stages of behavior change, health literacy, education, disease knowledge, and experience. To facilitate health behavior change, enable information seeking, and increase engagement, the educational materials provide different layers of information, including tips and information in the MEDSReM app, as well as more detailed educational content on the web portal. We will present examples of the materials in different formats to show how they are tailored to ease comprehension, support adherence, and influence behavior change. These educational materials will have broad utility outside of the MEDSReM system, and will also serve as the education-only comparison condition for the randomized controlled trial.

